# Membrane Charge Primes the Necroptotic Kinase RIPK3 for Amyloid Assembly

**DOI:** 10.1038/s42004-025-01658-0

**Published:** 2025-08-21

**Authors:** Fátima C. Escobedo-González, Andrea Gelardo, Alexandra Reimers, Paula Polonio, Miguel Mompeán, Gustavo A. Titaux-Delgado

**Affiliations:** 1 Instituto de Química Física Blas Cabrera, Consejo Superior de Investigaciones Científicas (IQF-CSIC), Serrano 119, 28006 Madrid, Spain; 2 Universidad Autónoma de Madrid, Escuela de Doctorado, Francisco Tomás y Valiente 2, 28049, Madrid, Spain

## Abstract

Receptor-interacting protein kinase 3 (RIPK3) drives necroptosis by assembling into functional amyloid fibrils. Here we show that lipids modulate RIPK3 amyloidogenesis by stabilizing an aggregation-prone intermediate. While electrostatic repulsion maintains RIPK3 in a soluble state, charge compensation alone is not sufficient for fibril formation and hydrophobic contacts are required to initiate nucleation. Using solution-state NMR, fluorescence-based assays and polymer-encased lipid particles, we demonstrate that negatively charged membranes selectively recruit RIPK3 and restrict its conformational flexibility, accelerating aggregation. These findings reveal a membrane-guided mechanism for RIPK3 assembly and suggest that lipid surfaces, like those implicated in pathological amyloid formation, may modulate functional amyloidogenesis even in the absence of canonical necroptotic stimuli.

## Introduction

Receptor-interacting protein kinase 3 (RIPK3) is a key regulator of necroptosis, a lytic form of programmed cell death distinct from apoptosis and essential for host defense.^1^ Unlike apoptosis, necroptosis relies on the transient assembly of RIPK3 into functional amyloid fibrils that act as scaffolds for downstream signaling. The intermolecular assembly of RIPK3 molecules is mediated by a conserved RIP homotypic interaction motif (RHIM).^2^ Once assembled into amyloids, RIPK3 phosphorylates mixed lineage kinase domain-like protein (MLKL), whose oligomerization at the plasma membrane compromises bilayer integrity and leads to cell lysis.^3,4^ Although tightly regulated under normal conditions, aberrant necroptosis (whether excessive or impaired) has been implicated in cancers, inflammatory diseases, and neurodegeneration. This underscores the need to understand the determinants of RIPK3 assembly.^5–7^

Amyloidogenesis, whether functional or pathological, often involves intrinsically disordered proteins (IDPs) adopting ordered fibrillar states. Beyond amyloid formation, many IDPs can interact with lipid membranes, a property that can significantly influence their conformational dynamics and assembly.^8–11^ Accordingly, growing in vitro evidence suggests that lipid bilayers modulate the aggregation of several amyloid-forming IDPs involved in neurodegeneration, such as Tau, Aβ, and α-synuclein.^12–16^ The physiological relevance of these membrane-induced amyloid fibrils and oligomers remains under active investigation.^15,17^

Unlike pathological amyloids such as Tau, Aβ, or α-synuclein that progressively deposit in the brain during neurodegeneration, functional amyloids are tightly regulated and efficiently cleared to prevent persistent accumulation.^18,19^ Notably, although it forms a functional amyloid, the RHIM of RIPK3 exhibits aggregation-prone characteristics akin to Tau^20^ and Aβ.^21^ We hypothesize that lipid membranes might similarly influence RIPK3 amyloidogenesis even in the absence of canonical necroptotic stimuli. If so, aberrant membrane-triggered assembly could lead to dysfunctional necroptosis and unwarranted cell loss, linking RIPK3 assembly to disease states.^5–7^ In other words, membranes could facilitate RIPK3 assembly and subsequent MLKL activation outside of classical necroptotic pathways, contributing to unintended necroptotic signaling as observed in inflammation or neurodegeneration. ^5–7^

## Results and Discussion

**Fig 1:**
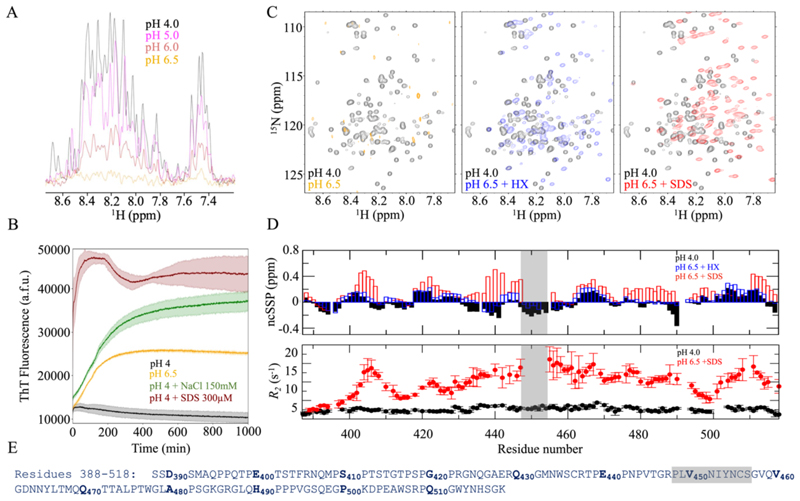
Charge compensation triggers CTD-RIPK3 self-assembly, while hydrophobic shielding prevents aggregation. **a** Solution-state 1D traces extracted from ^1^H–^15^N HSQC spectra reveal progressive signal loss as pH increases from 4.0 to 6.5, consistent with reduced electrostatic repulsion and a shift from monomeric to aggregated states. **b** ThT fluorescence assays show that increasing the pH, or adding 150 mM NaCl or 300 μM SDS (included as positive controls for promoting protein aggregation) at pH 4.0, induces rapid aggregation without a detectable lag phase. In contrast, pH 4.0 alone preserves a non-aggregated state. Error bars represent the standard deviation from three independent measurements **c** ^1^H–^15^N HSQC overlays comparing pH 4.0 (black spectrum) with pH 6.5 alone (orange spectrum) or in the presence of 10% 1,6-hexanediol (HX, blue) or SDS above the critical micelle concentration (red spectrum). HX and micellar SDS were included to probe conformational and dynamic changes under conditions known to modulate protein interactions and solubility. Addition of HX or SDS results in the recovery of sharp, well-dispersed peaks, indicating that both conditions prevent aggregation despite charge compensation at pH 6.5. **d** Top: neighbor-corrected secondary structure propensities (ncSSP) shows no specific tendency towards adopting structured ensembles at pH 4.0 or in the presence of 1,6-hexanediol, but helical propensity in the presence of SDS, which induces line broadening within the RHIM residues, consistent with specific micelle binding. Bottom: ^15^N *R*_*2*_ relaxation rates show elevated values in SDS, suggesting reduced mobility due to micelle association. Error bars represent fitting errors (1σ) obtained from mono-exponential fitting of the signal decay curves. **e** CTD-RIPK3 sequence with residues undergoing line broadening beyond detection are shaded gray.

To test whether lipid membranes influence RIPK3 assembly, we first investigate the factors governing its transition from a soluble IDP to amyloid fibrils in solution. For this purpose, we employed a truncated construct comprising the C-terminal domain of RIPK3 (residues 387–518), commonly referred to as CTD-RIPK3, which is widely used in RHIM assembly studies,^2, 20–24^ we first monitored its aggregation via solution NMR spectroscopy and Thioflavin-T (ThT) fluorescence. At pH 4.0 in the absence of salt, RIPK3 remained soluble and monomeric (calculated net charge ca. +8.8). Gradual neutralization of the solution led to a corresponding loss of NMR signal intensity, and by pH 6.5 (net charge ca. +2.0), all peaks in the spectrum had disappeared (Figure 1A and S1), indicating quantitative conversion of the protein into large, NMR-invisible aggregates. We therefore define pH 4.0 as a “non-assembling” condition, under which NMR signals remain stable for at least 48h without detectable decay (Figure S2).

Consistent with amyloid formation, ThT assays showed that neutralizing CTD-RIPK3’s positive charge accelerates fibrillization. Elevating the pH, or adding 150 mM NaCl or submicellar SDS at pH 4.0, where no assembly is expected, each produced a rapid increase in ThT fluorescence with essentially no detectable lag phase (Figure 1B). In contrast, the protein remained ThT-silent at pH 4.0 alone. Thus, diminishing electrostatic repulsion immediately triggers CTD-RIPK3 amyloid assembly without any detectable kinetic barrier under these conditions.

To probe whether hydrophobic interactions drive this aggregation, we introduced 10% 1,6-hexanediol, a disruptor of weak hydrophobic contacts. Remarkably, even at pH 6.5 this treatment preserved CTD-RIPK3 in solution, yielding a well-dispersed HSQC spectrum (Figure 1C). Similarly, adding SDS at millimolar concentrations (above its critical micelle concentration) prevented aggregation (Figure 1C). In both cases, the number of peaks in the HSQC was largely consistent with those of the monomeric pH 4.0 sample, indicating that a large fraction of the protein remained solubled and non-aggregated at pH 6.5, where the protein would otherwise assemble (Figure 1C).

We next compared the conformational ensembles under these protective conditions. Neighbor-corrected secondary structure propensities (ncSSP)^25^ displayed values that are typical of disordered conformations at pH 4.0, which were similar in the presence of 1,6 hexanediol. This indicates that the CTD-RIPK3 does not adopt major conformations under these conditions. However, in the presence of SDS, tendency towards nascent helical conformations were observed (Figure 1D, Table S1), along with larger localized perturbations consistent in signal broadening in the RHIM region, suggesting specific binding to the micelle (Figure 1D, Figure S3). This propensity of SDS micelles to induce helical structure in amyloid-forming proteins is consistent with previous reports.^26,27^ Along this line, backbone ^15^N *R*_2_ relaxation rates, which are sensitive to molecular dynamics, were markedly elevated in the presence of SDS, consistent with a micelle-bound state in which the protein’s motion is substantially restricted (Figure 1D).

Overall, these results indicate that CTD-RIPK3 aggregation is governed by both electrostatic and hydrophobic forces. Neutralizing the protein’s charge (by pH elevation or salt) is necessary to trigger aggregation, but hydrophobic contacts are required to nucleate and propagate the amyloid. Conversely, conditions such as SDS and hexanediol can suppress fibrillization by shielding hydrophobic surfaces or disrupting hydrophobic contacts, highlighting the dual regulation of amyloid assembly by charge and hydrophobicity. Notably, SDS micelles have been widely used as membrane mimetics in studies of amyloidogenic proteins including Tau, Aβ, or α-synuclein.^28–30^ Inspired by the ability of detergent micelles to bind and shield CTD-RIPK3, we next examined this intermolecular recognition in the context of more reliable membrane mimetics.

**Fig 2:**
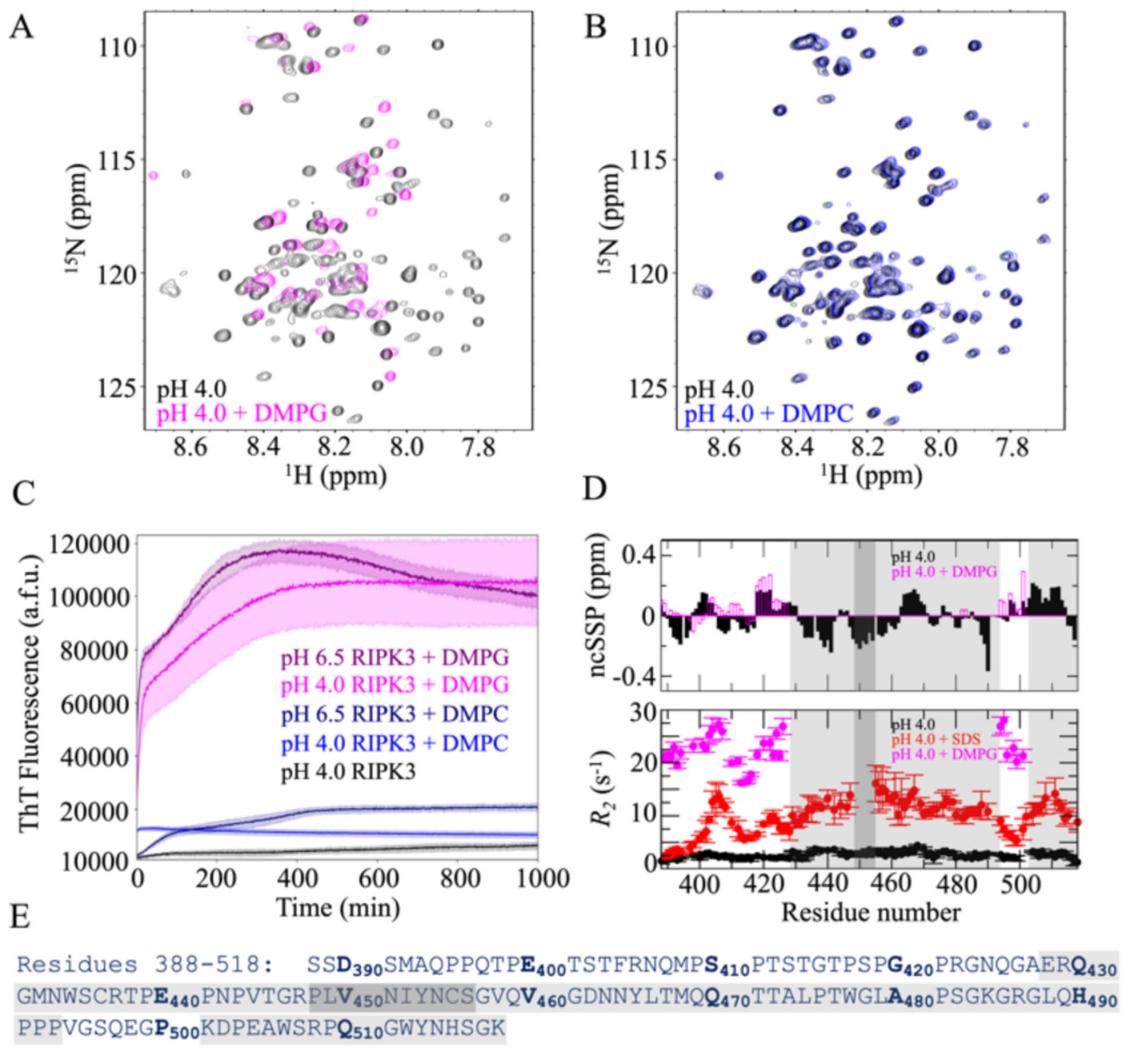
Membrane charge dictates CTD-RIPK3 interaction and aggregation kinetics **a** Overlay of ^1^H-^15^N HSQC spectra of CTD-RIPK3 at pH 4.0 in the absence (black) and presence (magenta) of negatively charged (DMPG) DIBMALPs. Multiple signals exhibit chemical shift perturbation or broaden/disappear, particularly for residues in the RHIM and flanking regions, consistent with strong interaction and altered dynamics. **b** Overlay of ^1^H-^15^N HSQC spectra at pH 4.0 in the absence of DIBMALPs (black) and in the presence of neutral (DMPC) DIBMALPs (blue). No signal changes are observed, indicating that electrostatic attraction is required for CTD-RIPK3-membrane interactions. **c** Aggregation kinetics monitored by ThT fluorescence at pH 4.0 and pH 6.5, with and without negatively (magenta and pink, respectively) or neutral (blue and navy, respectively) DIBMALPs. Negatively charged DIBMALPs accelerate aggregation even under non-assembling conditions at pH 4.0, while neutral DIBMALPs have no effect unless the pH is raised. Error bars represent the standard deviation from three independent measurements **d** Neighbor-corrected secondary structure propensities (ncSSP, top) and ^15^N *R*_*2*_ relaxation rates (bottom) for CTD-RIPK3 bound to negatively charged DIBMALPs. While no stable secondary structure is detected, the widespread increase in *R*_*2*_ values indicates strong DIBMALP binding and reduced conformational freedom across the sequence. Signals that disappear upon binding are shaded in gray. Note that the SDS-interacting region is encompassed within the lipid-interacting region. The corresponding error estimates for the ^13^Cα chemical shifts are reported in Table S2. For the relaxation data, error bars represent fitting errors (1σ) obtained from mono-exponential fitting of the signal decay curves. **e** CTD-RIPK3 sequence with residues undergoing line broadening beyond detection are shaded dark gray (SDS) and light gray (DIBMALPs).

To investigate how bona fide lipid membranes influence CTD-RIPK3 assembly, we employed polymer-encased lipid particles (DIBMALPs) containing either anionic,2 with Dimyristoyl-sn-glycero-3-phosphoglycerol (DMPG) or zwitterionic with 1,2-Dimyristoyl-sn-glycero-3-phosphocholine (DMPC) lipids. Unlike traditional membrane-scaffold protein nanodiscs, DIBMALPs remain stable at pH 4.0 (Figure S4), a condition under which CTD-RIPK3(387–518) is soluble and monomeric (Figure 1 and S2). Upon adding negatively charged DMPG DIBMALPs to CTD-RIPK3 at pH 4.0, we observed dramatic changes in the HSQC spectrum: numerous peaks broadened or disappeared, particularly those from the RHIM motif and immediately flanking residues (Figure 2A). This selective signal loss is a hallmark of membrane binding and closely mirrored the effects of SDS micelles, which broaden the same set of residues, indicating an overlapping binding interface. Notably, the pattern of perturbations was maintained when the pH was raised to 6.5 (Figure S5A and S6), demonstrating that the binding interface persists even under aggregation-prone conditions. By contrast, neutral DMPC produce no spectral changes (Figure 2B and S5B), confirming that electrostatic attraction is required for CTD-RIPK3 to bind the membrane surface.

To test whether membrane binding promotes amyloid assembly, as seen in pathological amyloids, we monitored CTD-RIPK3 fibrillization by ThT fluorescence at pH 4.0, which represent non-assembling conditions, and at pH 6.5 (Figure 2C). Negatively charged DMPG DIBMALPs markedly accelerated the increase in ThT signal under all conditions, even at pH 4.0 where CTD-RIPK3 alone does not aggregate. In contrast, neutral DMPC DIBMALPs had no apparent effect on the ThT kinetics relative to buffer, allowing fibril formation only when the solution was neutralized to pH 6.5 (Figure 2C). This selective acceleration of aggregation by anionic membranes indicates a membrane-induced nucleation mechanism.

We then examined the structural and dynamic properties of the membrane-bound state. The N- and C-terminal regions of CTD-RIPK3 remained largely disordered upon binding to anionic DIBMALPs. Δ^13^Cα secondary chemical shifts were minimal across most of the sequence, indicating no formation of stable secondary structure in these regions upon binding (Figure 2D, Table S1). However, membrane binding had a pronounced effect on protein dynamics: ^15^N *R*_*2*_ relaxation rates increased substantially for residues across the protein when bound to DMPG DIBMALPs (Figure 2D). This global ^15^N *R*_*2*_ elevation reflects reduced backbone mobility, consistent with RIPK3 being partially immobilized on the lipid surface. Notably, SDS micelles induced a similar relaxation profile, reinforcing a common binding mode. Thus, the lipid-bound protein still contains largely disordered regions but is conformationally restricted and significantly less dynamic than the free monomer. We infer that the lipid surface sequesters aggregation-prone segments (e.g., the RHIM) in a partially immobilized, exposed conformation that is especially susceptible to nucleation and fibril growth.

**Fig 3:**
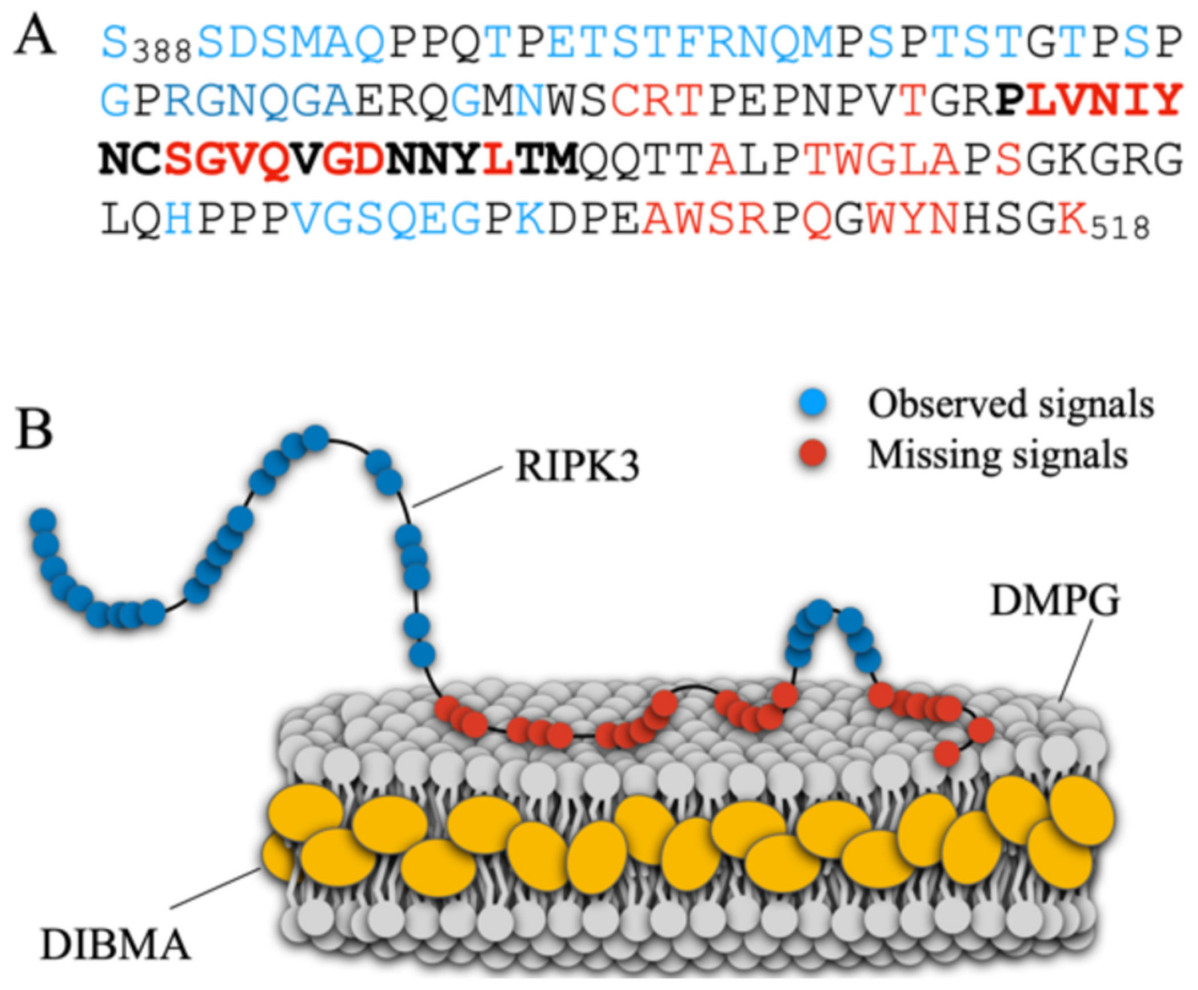
Lipid particles stabilize an intermediate state that primes CTD-RIPK3 for amyloid formation **a** Sequence of CTD-RIPK3 showing residues that do interact (red) or do not interact (blue) with DIBMALPs. Residues in black could not be unambiguously assigned. The RHIM core motif is indicated in bold. **b** Schematic model illustrating how negatively charged DIBMALPs recruit soluble CTD-RIPK3 into an intermediate, membrane-bound state. The DIBMALP surface binds aggregation-prone regions such as the RHIM and flanking segments, promoting its exposure and facilitating further nucleation and fibril growth.

Collectively, our findings demonstrate that membrane charge is a critical determinant of CTD-RIPK3’s aggregation pathway. Neutral DIBMALPs, lacking surface net charge have little impact on fibril formation. Negatively charged DIBMALPs, however, engage RIPK3 and promote a conformationally constrained membrane-bound intermediate that likely arises from monomeric RIPK3 and primes the protein for amyloidogenesis. This membrane-bound intermediate is distinct from the fully disordered, soluble monomer present at pH 4.0 (or in hexanediol at pH 6.5) and from the “shielded” state induced by SDS micelles; it represents a unique, nucleation-competent conformation. Based on these insights, we propose a membrane-guided model for CTD-RIPK3 amyloid assembly in which anionic lipid surfaces create an electrostatically tethered intermediate with reduced flexibility (Figure 3). This membrane-bound state poises CTD-RIPK3 for rapid assembly, allowing the bound monomers to nucleate and elongate into amyloid fibrils. Such a mechanism aligns with emerging evidence that pathological amyloid formation can be initiated at lipid interfaces.^12–16^ Overall, our results reveal a potential regulatory role for membrane lipids in the assembly of functional amyloids, shedding light on how cellular membranes may modulate CTD-RIPK3’s necroptotic signaling.

## Methods

### Protein Expression and Purification

The human CTD-RIPK3 (387–518) construct, codon-optimized for *Escherichia coli* expression, was purchased from GenScript (New Jersey, NJ, USA) and subcloned into a pET11a-derived vector containing an N-terminal His_6_-tag followed by a tobacco etch virus (TEV) protease cleavage site. The resulting fusion protein was expressed in *E. coli* BL21 (DE3) cells. Transformed *E. coli* cells were initially cultured in 2 L of 2×YT medium at 37°C until an optical density at 600 nm (OD_600_) of 0.6–0.8 was reached. Cells were harvested by centrifugation at 4,000 × g for 15 minutes at 4°C, and the resulting pellet was resuspended in 0.5 L of M9 minimal medium supplemented with ^13^C-glucose and ^15^NH_4_Cl (Cambridge Isotope Laboratories) as the sole carbon and nitrogen sources, respectively. To ensure efficient isotopic labeling, cells were incubated for 1.5 hours at 37°C prior to induction. Protein expression was induced by addition of 0.5 mM IPTG, followed by overnight incubation at 25°C. For protein purification, harvested cells were resuspended in lysis buffer containing 20 mM Tris-HCl, 150 mM NaCl, and 1 mM EDTA (pH 8.0), and lysed by sonication on ice in 45-second bursts for 3 to 5 cycles. The lysate was clarified by centrifugation at 20,000 rpm for 20 minutes at 4°C, and the insoluble fraction containing inclusion bodies was collected. Inclusion bodies were solubilized in a buffer containing 6 M guanidine hydrochloride (GuHCl), 20 mM Tris-HCl (pH 8.0), and 2 mM DDT. The His-tagged protein was purified under denaturing conditions using a Ni^2+^-affinity chromatography column 5mL (HiTrap, Cytiva, Freiburg, Germany).

The Ni^2+^-affinity column was washed with a buffer containing 8 M urea, 50 mM Tris-HCl, and 1 mM DTT (pH 8.0). Bound protein was eluted using the same buffer supplemented with 500 mM imidazole, also adjusted to pH 8.0. The eluted protein solution was passed through a 0.22 μm membrane filter and subsequently subjected to size-exclusion chromatography (SEC) using a Superose 6 Increase 10/300 GL column (Cytiva, Freiburg, Germany) connected to an ÄKTA FPLC system (Cytiva, Freiburg, Germany), to separate monomeric protein from higher-order oligomeric species.

### Preparation of DIBMALPs

1,2-dimyristoyl-sn-glycero-3-phosphocholine, DMPC and 1,2-dimyristoyl-sn-glycero-3-phospho-(1'-rac-glycerol), DMPG lipid (Avanti Polar Lipids) powders were individually resuspended in phosphate buffer (20 mM Na_2_HPO_4_/NaH_2_PO_4_, pH 6.5) to a final concentration of 20 mM. Each lipid suspension was thoroughly mixed by vortexing and equilibrated at 30°C for 15 minutes. To promote the formation of unilamellar structures, the suspensions underwent nine freeze–thaw cycles by alternating immersion in liquid nitrogen and a 60°C water bath. Large unilamellar vesicles (LUVs) were then prepared by extruding each lipid suspension 30 times through a 100 nm polycarbonate membrane using a manual extruder (Avanti Polar Lipids, Germany).

Lyophilized diisobutylene/maleic acid copolymer (DIBMA) was purchased from Cube Biotech (Monheim am Rhein, Germany) and reconstituted in phosphate buffer (20 mM Na_2_HPO_4_/NaH_2_PO_4_, pH 6.5) to a final concentration of 100 mg/mL. The polymer solution was then added gradually to the LUV suspension to obtain a final lipid-to-polymer mass ratio of 1:1. The resulting mixture was incubated at 30°C for at least 1 hour to initiate DIBMALPs assembly. To ensure complete formation and stabilization, samples were subsequently maintained at 4°C for 16 hours. Insoluble aggregates were removed by centrifugation at 15,000 × g for 30 minutes at 4°C.

To further purify the preparations and separate potential aggregates or polymer excess, samples were subjected to size-exclusion chromatography (SEC). A Superdex 200 Increase 10/300 GL column (Cytiva, Freiburg, Germany), connected to an ÄKTA purifier system (Cytiva, Freiburg, Germany), was equilibrated with two column volumes of 20 mM Na_2_HPO_4_/NaH_2_PO_4_ buffer at pH 6.5. DIBMALPs sample was loaded onto the column and eluted at a flow rate of 0.5 mL/min.

### Thioflavin T fluorescence assays of amyloid assembly

To monitor amyloid formation under different experimental conditions, including pH variation and the presence of SDS, NaCl, or DIBMALPs, fluorescence assays with Thioflavin T (ThT) were performed. The assembly buffer contained 1 mM acetic acid, 1 mM TCEP, and 40 μM ThT, and its pH was adjusted to either 4.0 or 6.5 by stepwise addition of 100 mM Na_2_CO_3_. SEC-purified RIPK3 was maintained in its monomeric form in a denaturing buffer containing 8 M urea and 1 mM TCEP, pH 4.0, at a stock concentration of 200 μM. NaCl (150mM) and SDS submicellar (300uM) were included as positive controls for promoting protein aggregation. To initiate amyloid formation, aliquots of the monomeric CTD-RIPK3 stock were transferred into black half-area 96-well flat-bottom polystyrene microplates (Thermo Scientific™ Nunc™, Cat. No. 167008), Nunclon™ Delta-treated, suitable for fluorescence measurements. Assembly was triggered by rapidly diluting the urea concentration to 100 mM through the addition of an assembly buffer pre-adjusted. This dilution also brought the final concentration of CTD-RIPK3 to 5 μM. All reactions were performed at room temperature.

Fluorescence was recorded immediately after mixing using a POLARstar Omega microplate reader (BMG Labtech) with excitation and emission filters set at 440 ± 10 nm and 480 ± 10 nm, respectively. Assays were performed in triplicate, and fluorescence data were collected every 30 seconds over a 20-hour period. The final protein concentration in each well was 5 μM, determined by absorbance at 280 nm using a NanoDrop™ 2000C spectrophotometer (Thermo Fisher Scientific, Scoresby, VIC, Australia).

### NMR spectroscopy

Nuclear magnetic resonance (NMR) experiments were conducted at 298 K on a Bruker Avance Neo 800 MHz spectrometer (^1^H frequency), equipped with a TCI cryoprobe and Z-gradient capabilities. All measurements were performed using uniformly ^13^C,^15^N-labeled RIPK3 protein samples. Prior to acquisition, samples were desalted into a buffer containing 90:10 H_2_O/D_2_O, supplemented with 1 mM acetic acid (pH adjusted to 4.0) and 1 mM TCEP. For pH titration experiments, the pH was incrementally raised to 5.0, 5.5, and 6.5 by the addition of small aliquots (0.5 μL) of 100 mM Na_2_CO_3_. Protein concentrations were estimated to be between 20 and 80 μM by measuring UV absorbance at 280 nm. Depending on the experimental condition, SDS, 1,6-hexanediol, or DIBMALPs were subsequently added to the samples to evaluate their effect on amyloid assembly under different environments. In particular, studies with DIBMALPs were conducted at a protein concentration 20-22 μM. Experiments in the presence of 1,6-hexanediol and SDS micelles were carried out at protein concentrations of 50 and 80 μM, respectively.

Sequential backbone assignments were initiated using H(NCOCA)HN and (H)N(COCA)NH experiments, each acquired with 32 scans. Spectral widths and transmitter offsets were 12/20/4.70 ppm and 4.70/117/4.70 ppm (^1^H/^15^N/^1^H) for H(NCOCA)HN, and 12/20/20 ppm and 4.70/117/117 ppm (^1^H/^15^N/^15^N) for (H)N(COCA)NH. Spin system connectivity was then established using standard ^13^C-based triple-resonance experiments: HNCA (^1^H/^15^N/^13^C Spectral widths= 12/20/30 ppm; offsets = 4.70/117/53.2 ppm), CBCA(CO)NH (12/20/80 ppm; 4.70/117/43 ppm), HNCACB (9.7/20/80 ppm; 4.70/117/39.5 ppm), HNCO (12/20/12 ppm; 4.70/117/173.5 ppm), and HNCACO (9.7/23/16 ppm; 4.70/117/173.5 ppm). All experiments were acquired with 32 scans, except HNCO, which was acquired with 8 scans. Complete backbone assignments were achieved for all residues except prolines, which lack amide protons and are therefore not observable in these spectra.

All datasets were processed using TopSpin 4.1.8 (Bruker Biospin, Germany), and resonance assignments were performed with NMRFAM-Sparky. Proton chemical shifts were referenced directly to DSS, while ^13^C and ^15^N chemical shifts were referenced indirectly.

Longitudinal (^15^N *R*_*1*_) relaxation rates were measured on a (^15^N -labelled diluted sample (30 μM) using the standard Bruker pulse sequence hsqct1etf3gpsi, with relaxation delays of 8, 100, 240, 460, 800, and 1200 ms. Spectra were acquired with 8 scans, using spectral widths of 12 ppm for ^1^H and 20 ppm for ^15^N, and transmitter frequency offsets of 4.70 ppm and 117 ppm for ^1^H and ^15^N, respectively. Rotating-frame (^15^N *R*_1_ρ) relaxation rates were determined using the standard Bruker pulse sequence hsqctref3gpsi with relaxation delays of 8, 36, 76, 100, 156 and 300 ms, and a spin-lock field strength of 2 kHz. Spectra were recorded with 16 scans using spectral widths of 12 ppm for ^1^H and 20 ppm for ^15^N, and transmitter offsets of 4.70 and 117 ppm, respectively. ^15^N *R*_*2*_ were obtained from *R*_1_ρ and *R*_1_ values using the relation *R*_1_ρ = *R*_1_ cos^2^*θ* + *R*_2_ sin^2^*θ*, where *θ* is the tilt angle between the laboratory z-axis and the spin-lock axis defined by the amplitude of the applied spin-lock field ω_1_ and the offset Ω, that is, *θ* = arctan (ω_1_/Ω), following the definitions in Palmer, A. G., and Massi, F. ^31^

Chemical shift perturbation (CSP) values were calculated using the combined chemical shift difference formula: CSP = [(Δδ^1^H)^2^+(α·Δδ^15^N)^2^]^1/2^, where ΔδH and ΔδN are the changes in amide proton and nitrogen chemical shifts (in ppm), respectively, with α = 0.154 to account for the difference in the spectral widths of ^1^H and ^15^N dimensions.^32^

## Supplementary Material

Supplementary Information

## Data Availability

The authors declare that the data supporting the findings of this study are available within the paper and its Supplementary Information files. Should any raw data files be needed in another format they are available from the corresponding author upon reasonable request.
