# Supplementary material for: Membrane Charge Primes the Necroptotic Kinase RIPK3 for Amyloid Assembly
Source: Commun Chem. Author manuscript; Available in PMC 2025 Sep 9. (PMC12370984; doi:10.1038/s42004-025-01658-0)
Supplement: Supplementary Information [file EMS207812-supplement-Supplementary_Information.pdf]

Supporting Information for:

Membrane Charge Primes the Necroptotic Kinase RIPK3 for Amyloid Assembly

Fátima C. Escobedo-González[1], Andrea Gelardo[1], Alexandra Reimers[1], Paula Polonio[1,2], Miguel Mompeán[1*], Gustavo A. Titaux-Delgado[1*]

[1]Instituto de Química Física Blas Cabrera, Consejo Superior de Investigaciones Científicas (IQF-CSIC), Serrano 119, 28006 Madrid, Spain.
[2] Universidad Autónoma de Madrid, Escuela de Doctorado, Francisco Tomás y Valiente 2, 28049, Madrid, Spain.

*Correspondence: mmompean@iqf.csic.es, gtitaux@iqf.csic.es

Contents

-Supplementary Figures

Figure S1. Charge compensation triggers CTD-RIPK3 self-assembly.

Figure S2. ^{1}H NMR spectra of RIPK3 at pH 4.0 recorded at different time points (0, 12, 24 and 48 hours) at 298 K.

Figure S3. Relative intensity ratios per residue comparing signal attenuation in the presence of 1,6-hexanediol (HX) and SDS with respect to the pH 4.0 condition.

Figure S4. Negatively charged DIBMALPs remain stable at pH 4.0.

Figure S5. RIPK3 interacts strongly with negatively charged, but not so with neutral, DIBMALPs at pH 6.5

Figure S6. Residue-specific chemical shift perturbation (CSP) values comparing spectra acquired at pH 4 and pH 6.5, both in the presence of DIBMALPs

Table S1. Chemical shift assignments of RIPK3 under different experimental conditions.

Table S2. Secondary Cα chemical shifts of RIPK3 with corresponding error estimates based on signal-to-noise ratio analysis.

Figure S1

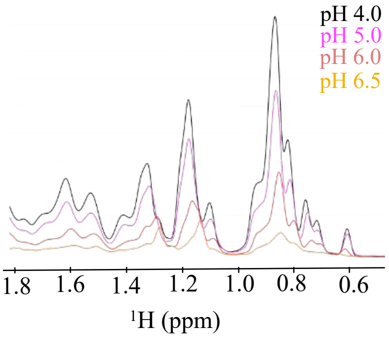

Figure S1. Charge compensation triggers CTD-RIPK3 self-assembly. Solution-state ^{1}H NMR spectra (methyl region, 0.6–1.8 ppm) show progressive signal loss as pH increases from 4.0 to 6.5, consistent with quantitative conversion of the protein into large, NMR-invisible aggregates.

Figure S2

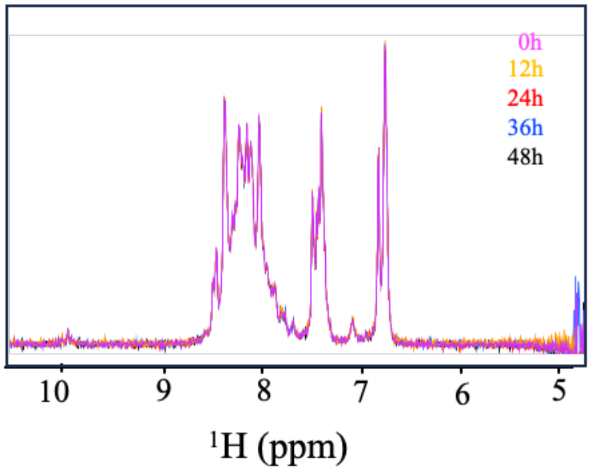

Figure S2. ^{1}H NMR spectra of RIPK3 at pH 4.0 recorded at different time points (0, 12, 24 and 48 hours) at 298 K.

The spectra show no detectable loss of signal intensity or line broadening over this period, indicating that the protein remains monomeric and stable under these conditions. Spectra are color-coded as follows: 0 h (magenta), 12 h (yellow), 24 h (red), 36 h (blue), and 48 h (black).

Figure S3

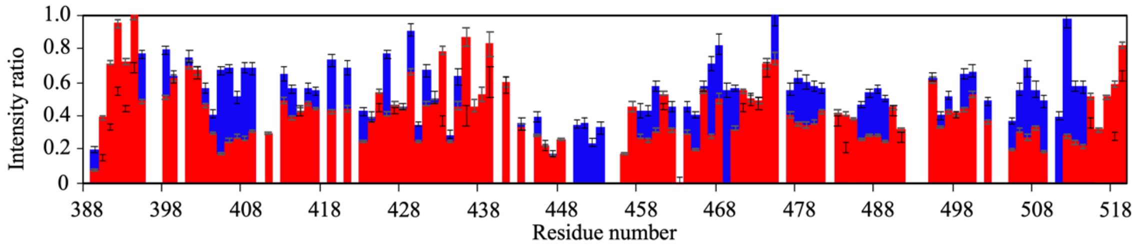

Figure S3. Relative per residue intensity ratios.
Signal attenuation upon raising the pH from 4.0 to 6.5 in the presence of 1,6-hexanediol (HX, blue), and SDS (red) with respect to the pH 4.0 condition.

Figure S4

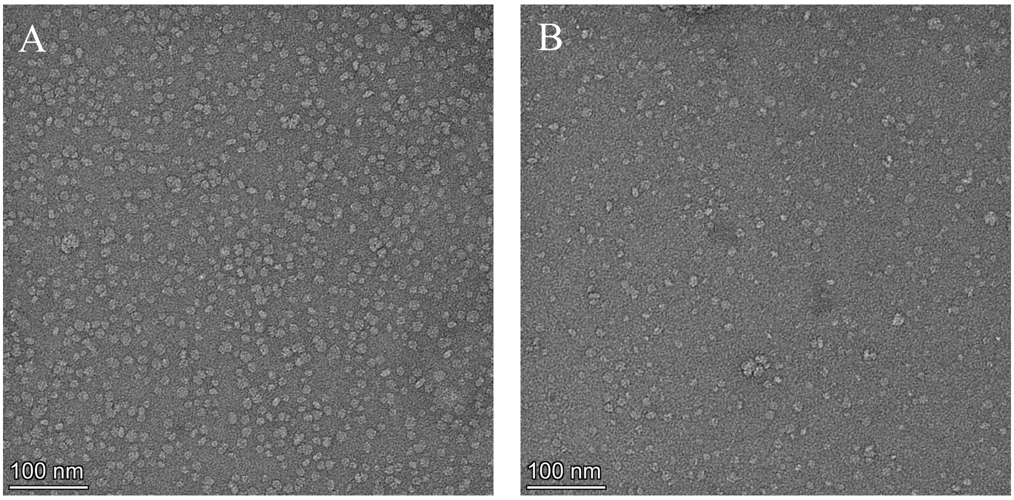

Figure S4. Negatively charged DIBMALPs remain stable at pH 4.0.
Transmission electron microscopy (TEM) images of DMPG-based DIBMALPs at pH 4.0 (**A**) before and (**B**) after addition of RIPK3(387–518).

Figure S5

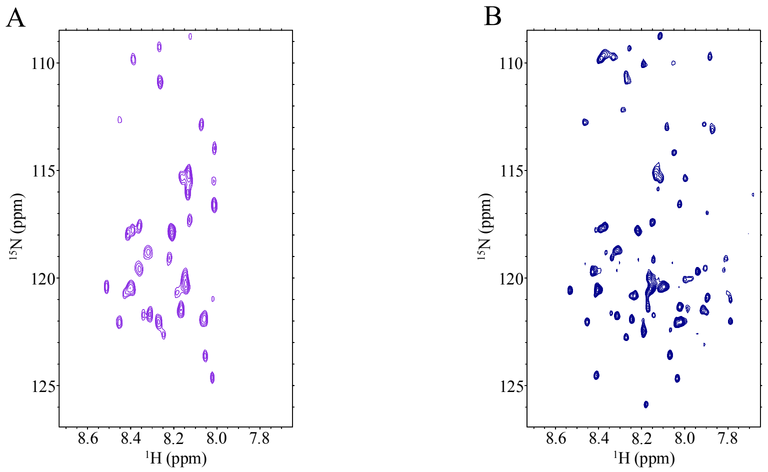

Figure S5. RIPK3 interacts strongly with negatively charged, but not so with neutral, DIBMALPs at pH 6.5.
^{1}H–^{15}N HSQC spectra of RIPK3(387–518) in the presence of **(A)** negatively charged DMPG DIBMALPs (violet) or **(B)** neutral DMPC DIBMALPs (dark blue) at pH 6.5. Related to Figure 2 in the main text.

Figure S6

A

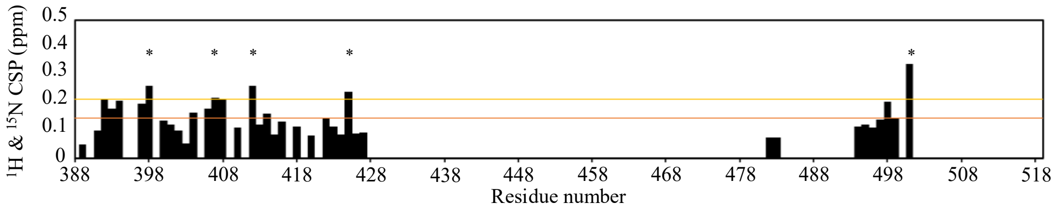

B

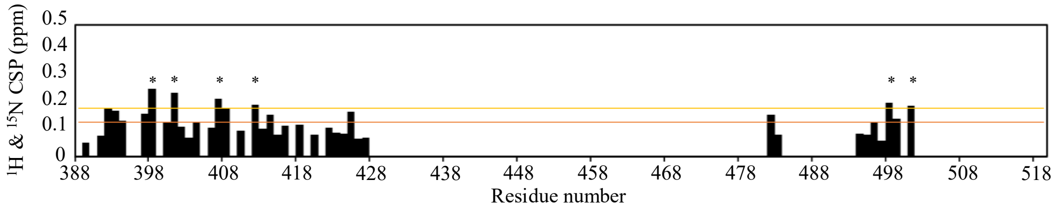

C

Residues 388-518: SS**D**$_{390}$SMAQPPQT*PE*$_{400}$TSTFRNQ*MP**S**$_{410}$PT*STGTPSP**G**$_{420}$PRGNQ*GAER**Q**$_{430}$
GMNWSCRTP**E**$_{440}$PNPVTGRPL**V**$_{450}$NIYNCSGV*Q**V**$_{460}$GDNNYLTMQ**Q**$_{470}$TTALPTWGL**A**$_{480}$PSGKGRGLQ**H**$_{490}$
PPPVGSQEG**P**$_{500}$KDPEAWSRP**Q**$_{510}$GWYNHSGK*

Figure S6. ^{1}H and ^{15}N chemical shift perturbation (CSP) values comparing spectra acquired at pH 4 and pH 6.5, both in the presence of DIBMALPs.

Per residue ^{1}H-^{15}N CPS values for ^{15}N-CTD-RIPK3 in the presence of DMPG DIBMALPs at 25°C and pH 4.0 (**A**) and 6.5 (**B**). The orange line represents the mean CSP value (μ) and the yellow line is the mean plus one standard deviation (μ + σ). The main chain ^{1}H–^{15}N signals with CSP values > (μ + σ) at pH 4.0 are T398, Q407, T412, Q425, and K501, and are marked with asterisks. At pH 6.5, T401 and E499 also showed CSP values > (μ + σ).

(**C**) CTD-RIPK3 sequence with residues undergoing line broadening beyond detection are shaded dark gray (SDS) and light gray (DIBMALPs), matching the color code shown in Fig. 2 in the main manuscript. Asterisks indicate residues with CSP values > (μ + σ) at pH 4.0.

Table S1 Chemical shift assignments of RIPK3 under different experimental conditions. ^{1}H, ^{15}N, Cα, and Cβ chemical shift assignments were obtained for pH 4.0, pH 6.5 with 5% 1,6-hexanediol, and pH 6.5 with 1% SDS samples. For the pH 4.0 condition in DIBMALPs, only ^{1}H, ^{15}N, and Cα assignments were obtained due to the absence of ^{13}C labeling in this condition.

	pH 4					SDS pH 6					Hexa pH 6 5					DibmaLPs pH4		
Assignmer	C	CA	CB	H	N	C	CA	CB	H	N	C	CA	CB	H	N	CA	H	N
G387	-	-	-	-	-	-	-	-	-	-	173,8	45,17	-	7,78	109,9	43,408	-	-
S388	174,8	58,54	63,65	8,67	120,7	-	58,77	63,66	8,64	115,84	174,8	58,16	64,07	8,12	115,3	58,431	8,765	115,64
S389	174,4	58,56	63,59	8,46	120	174,23	58,71	-	8,45	117,46	174,3	58,66	63,7	8,4	117,7	58,674	8,421	117,58
D390	176,1	54,11	40,09	8,35	124,5	176,28	54,5	40,95	8,15	121,69	176,4	54,54	41	8,23	121,9	-	-	-
S391	174,5	58,69	63,57	8,29	118,7	174,48	58,65	63,71	8,09	115,6	174,6	58,81	63,71	8,1	115,8	58,776	-	-
M392	175,8	55,35	32,61	8,33	124,4	175,65	55,35	32,72	8,18	121,57	175,7	55,41	32,57	8,16	121,3	55,447	8,21	121,43
A393	177,3	52,36	19,04	8,23	127,7	177,02	52,25	19,07	8,05	124,66	-	52,32	19,12	8	124,5	52,341	8,117	124,51
Q394	-	53,35	-	8,34	123,2	173,22	53,33	28,97	8,11	120,03	-	53,32	-	8,13	120,2	-	8,188	120,2
P395	-	-	-	-	-	-	-	-	-	-	-	-	-	-	-	-	-	-
P396	175,7	62,99	31,99	-	-	-	-	-	-	-	-	62,85	31,93	-	-	-	-	-
Q397	175,9	55,56	29,64	8,56	123,3	175,65	55,51	29,6	8,33	120,15	175,8	55,58	29,69	8,38	120,5	55,607	8,483	120,26
T398	-	59,75	-	8,32	120,5	175,65	59,84	69,53	8,09	116,95	-	59,59	-	8,12	117,2	59,731	8,16	117,35
P399	176,9	63,13	31,96	-	-	-	-	-	-	-	177	63,55	31,96	-	-	-	-	-
E400	176,6	56,33	29,24	8,6	123,1	176,57	56,72	29,82	8,45	120,55	176,9	57,12	29,93	8,54	120,4	56,025	-	-
T401	174,7	61,78	69,7	8,22	117,2	174,46	61,81	69,77	7,98	113,92	-	62,04	69,62	8,02	113,9	61,87	8,11	114,33
S402	174,8	58,51	63,68	8,4	120,4	174,49	58,36	63,69	8,21	118,08	174,8	58,73	63,67	8,19	117,7	58,531	8,312	117,8
T403	175,6	62,09	69,45	8,15	118,1	175,17	62,34	70,08	8,08	115,93	174,4	62,3	69,49	7,97	115,3	62,251	8,111	115,55
F404	174,4	57,89	39,27	8,2	124,9	176,7	60,12	39,12	8,13	121,23	175,6	57,99	38,99	8,01	121,8	58,101	8,167	121,72
R405	175,6	55,98	30,7	8,2	125	177	57,8	30,02	7,89	117,62	175,6	56,05	30,64	8	121,8	56,207	-	-
N406	174,8	53,24	38,62	8,36	122	175,3	54,2	38,81	7,85	116,25	174,8	53,38	38,62	8,18	119	53,398	8,285	118,79
Q407	175,5	55,65	29,56	8,33	122,8	175,31	55,39	29,51	7,77	117,36	175,4	55,66	29,6	8,14	119,8	55,713	8,191	119,83
M408	-	53,28	-	8,44	125,5	173,97	53,6	32,75	7,59	120,24	-	53,28	-	8,24	122,5	53,277	8,309	122,57
P409	176,6	63,05	31,98	-	-	-	-	-	-	-	176,5	63,03	31,93	-	-	63,147	-	-
S410	-	56,27	-	8,55	120,4	173,36	55,61	63,86	8,12	114,51	-	56,24	-	8,35	117,6	56,291	8,453	117,77
P411	177,1	63,31	32,12	-	-	-	-	-	-	-	177,1	63,49	32,12	-	-	63,579	-	-
T412	174,7	61,66	69,74	8,24	115,9	174,96	61,98	69,62	8	111,49	-	61,68	69,28	8,05	112,8	61,714	8,133	112,65
S413	174,8	58,23	63,78	8,38	120,6	-	58,62	63,74	8,08	117,32	174,7	58,23	63,79	8,18	117,7	58,386	8,264	117,88
T414	175	61,83	69,66	8,29	117,9	175,15	61,97	69,66	8,04	114,14	175	61,93	69,64	8,11	115	61,932	8,196	114,98
G415	173,8	45,12	-	8,42	113,6	173,59	45,13	-	8,17	110,78	173,7	45,11	-	8,25	110,7	45,127	8,331	110,96
T416	-	59,78	-	8,18	119,3	172,61	59,63	69,69	7,89	115,59	-	59,75	-	7,99	116,5	59,726	8,069	116,57
P417	176,6	62,96	32,04	-	-	-	-	-	-	-	176,5	62,88	32,02	-	-	62,936	-	-
S418	-	56,25	-	8,53	120,3	172,62	56	63,8	8,22	116,33	-	56,27	-	8,34	117,4	56,269	8,43	117,54
P419	177,1	63,28	32,15	-	-	-	-	-	-	-	176,9	63,36	32,02	-	-	63,354		
G420	-	44,41	-	8,27	111,5	172,23	44,49	-	7,99	108,04	-	44,43	-	8,1	108,6	44,453	8,195	108,85
P421	177,3	63,05	31,97	-	-	-	-	-	-	-	177,2	63,25	32,11	-	-	-	-	-
R422	176,9	56,04	30,65	8,57	123,5	177,04	56,61	30,16	8,15	118,49	176,8	56,02	30,88	8,4	120,6	56,285	8,508	120,68
G423	173,8	45,26	-	8,4	112	174,27	45,56	-	8,07	108	173,8	45,28	-	8,24	109,2	45,398	8,343	109,23
N424	175,5	53,04	38,72	8,47	121,4	175,38	53,41	38,81	8,08	118,28	-	53,13	38,8	8,31	118,6	53,207	8,384	118,75
Q425	176,5	56,24	29,08	8,55	123,3	176,54	56,48	28,78	8,32	119,56	176,4	56,32	29,2	8,38	120,4	56,257	8,459	120,31
G426	174	45,33	-	8,52	112,3	174,07	45,51	-	8,32	109,45	174	45,33	-	8,39	109,7	45,422	8,434	109,71
A427	177,9	52,6	19,21	8,2	126,2	177,6	52,61	19,31	7,9	123,24	177,9	52,69	19,2	8,06	123,5	52,54	8,114	123,51
E428	176,4	56,27	29,26	8,46	122,1	176,31	56,31	29,51	8,21	118,7	176,6	57,02	29,72	8,44	119,6	-	-	-
R429	176,1	55,87	30,38	8,33	124,3	-	56,19	30,46	8,08	121,64	176,1	56,04	30,47	8,16	121	-	-	-
Q430	176,4	56,02	29,3	8,41	123,7	176,32	56,32	29,48	8,31	121,17	176,3	56,09	29,28	8,21	120,7	-	-	-
G431	174,1	45,23	-	8,49	112,6	173,71	45,16	-	8,09	108,74	174	45,3	-	8,31	109,6	-	-	-
M432	175,9	55,4	32,44	8,23	122	175,64	55,32	32,74	7,91	118,9	175,7	55,45	32,76	8,04	119,2	-	-	-
N433	174,9	53,02	38,49	8,47	122	174,95	53,27	38,66	8,19	119,29	174,7	53,14	38,62	8,28	119,3	-	-	-
W434	-	57,43	29,52	8,14	124,3	176,5	57,92	29,64	7,84	121,38	176	57,41	29,68	7,98	121,5	-	-	-
S435	-	58,46	63,82	8,14	119,4	174,39	59,26	63,63	7,94	115,22	-	58,39	-	8,07	116,3	-	-	-
C436	-	58,36	27,77	8,18	122,9	174,29	58,75	27,67	7,78	118,86	-	-	-	-	-	-	-	-
R437	-	55,92	30,76	8,36	125,7	175,48	56,15	30,8	7,81	121,3	175,7	55,63	31,05	-	-	-	-	-
T438	-	59,74	-	8,23	120,7	172,69	-	69,45	7,94	115,29	-	59,84	-	8,08	117,6	-	-	-
P439	176,7	62,97	32,18	-	-	-	-	-	-	-	176,2	62,97	32,23	-	-	-	-	-
E440	-	53,8	-	8,46	124,9	-	56,63	29,5	8,04	120,58	-	54,17	-	8,28	122,3	-	-	-
P441	176,2	62,91	31,98	-	-	-	-	-	-	-	176,1	62,93	31,7	-	-	-	-	-
N442	-	51,24	-	8,54	122,6	173,26	51,43	39,08	8,31	119,19	-	51,16	-	8,29	119,8	-	-	-
P443	-	63,13	32,15	-	-	-	-	-	-	-	177,1	63,48	32,24	-	-	-	-	-
V444	176,6	62,8	32,35	8,33	122,7	176,4	63,23	-	8,16	118,97	176,8	63,44	32,4	8,12	118,7	-	-	-
T445	175	61,82	69,66	-	8,11	175,01	62,07	69,63	7,78	113,55	175,4	62,08	69,7	7,72	112,5	-	-	-
G446	173,5	45,11	-	8,42	113,6	-	45,24	-	8,09	110,56	-	45,32	-	8,28	110,7	-	-	-
R447	-	53,79	-	8,14	123,9	-	54,19	-	7,97	120,01	-	-	-	-	-	-	-	-
P448	176,6	62,85	32,01	-	-	-	-	-	-	-	176,6	63,04	32,11	-	-	-	-	-
L449	177,1	55,19	42,17	8,46	125,4	-	-	-	-	-	177	55,32	42,3	8,38	122,8	-	-	-
V450	175,3	61,72	32,85	8,06	122,8	-	-	-	-	-	175,2	61,88	-	7,87	119,2	-	-	-
N451	174,8	52,89	38,89	8,55	125,2	-	-	-	-	-	174,9	52,96	-	8,36	122,1	-	-	-
I452	175,6	61,31	38,42	8,03	122,7	-	-	-	-	-	-	61,52	-	7,8	119,4	-	-	-
Y453	-	57,62	38,65	8,25	125,6	-	-	-	-	-	-	-	-	-	-	-	-	-
N454	175	52,89	38,67	8,29	122,6	-	-	-	-	-	-	-	-	-	-	-	-	-
C455	-	58,81	27,81	8,39	121,9	-	-	-	7,98	117,33	-	-	-	-	-	-	-	-
S456	-	55,9	63,79	8,46	120,4	-	59,97	64,24	7,94	116,03	174,6	-	-	-	-	-	-	-
G457	173,8	45,3	-	8,36	113	-	45,55	-	7,9	109,68	173,7	45,27	-	8,27	110,4	-	-	-
V458	175,9	62,14	32,61	7,98	121,9	175,06	62,39	32,56	7,62	119,16	175,7	62,09	32,95	7,8	119	-	-	-
Q459	175,7	55,47	29,47	8,56	127,3	175,46	54,78	30,12	8,18	123,49	175,6	55,42	29,69	8,4	124,4	-	-	-
V460	176,5	62,39	32,56	8,31	124,5	-	-	32,72	8,05	120,74	176,3	62,41	32,43	8,14	121,5	-	-	-
G461	173,7	45,17	-	8,47	114,9	174,14	45,35	-	8,3	111,58	173,6	45,11	-	8,28	112,1	-	-	-
D462	175,8	53,65	40,26	8,29	122,5	175,55	54,32	40,52	8,1	119,66	176,2	54,23	41,26	8,08	120,3	-	-	-
N463	175,1	53,38	38,54	8,52	121,6	174,48	53,52	39,21	8,1	116,62	-	53,65	38,54	8,35	118,7	-	-	-
N464	175,2	53,55	38,53	8,42	121,4	175,2	53,85	38,79	8,17	117,73	175,3	53,77	38,54	8,3	118,7	-	-	-
Y465	176	58,6	38,18	8,14	122,7	176,26	59,81	-	8,1	119,04	176	58,85	38,22	8,01	119,9	-	-	-
L466	177,8	55,67	42,2	8,05	124,4	177,28	55,7	42,06	7,93	118,44	177,8	55,8	42,25	7,88	121,2	-	-	-
T467	175	62,29	69,5	8,02	115,9	175,36	62,4	69,85	7,79	112,1	175	62,4	69,64	7,84	112,9	-	-	-
M468	176,3	55,93	32,39	8,23	124,3	-	-	-	-	-	176,3	56,11	32,38	8,04	121,4	-	-	-
Q469	-	56,01	29,08	8,33	123,3	176,34	56,75	28,9	8,12	119,03	176	56,04	29,14	8,15	120,3	-	-	-
Q470	-	56,02	29,38	8,41	123,7	176,42	56,54	-	8,07	118,27	176,2	56,09	29,46	8,22	120,5	-	-	-
T471	174,6	62,13	69,64	8,29	117,7	174,65	62,39	69,52	7,89	112,14	174,5	62,07	69,66	8,1	114,6	-	-	-
T472	173,9	61,69	69,7	8,12	118,6	173,57	62,14	69,85	7,8	115,24	173,8	61,76	69,65	7,93	115,6	-	-	-
A473	177,1	52,1	19,16	8,28	129	176,64	51,94	19,63	7,93	125,23	177	52,14	19,27	8,09	126	-	-	-
L474	-	52,93	-	8,11	125,4	175,08	53,59	41,46	7,8	120,63	-	52,93	-	7,92	122,4	-	-	-
P475	177	63	31,75	-	-	-	-	-	-	-	176,9	62,99	31,67	-	-	-	-	-
T476	174,5	61,88	69,48	8,06	115,6	174,52	61,33	69,42	7,72	111,01	174,4	61,89	69,58	7,89	112,7	-	-	-
W477	176,5	57,12	29,4	7,94	124,7	176,49	57,59	29,68	7,75	122,51	176,4	57,23	29,48	7,77	121,9	-	-	-
G478	173,5	45,26	-	8,21	112,8	173,88	45,85	-	8,04	108,63	173,5	45,28	-	8,04	109,8	-	-	-
L479	176,7	54,6	42,36	7,94	123,7	176,24	55,04	42,64	7,56	120,06	176,6	54,65	42,45	7,77	120,9	-	-	-
A480	-	50,45	-	8,33	128,7	175,25	50,76	18,2	7,96	122,68	-	50,43	-	8,15	125,7	-	-	-
P481	177	63,14	31,94	-	-	-	-	-	-	-	176,9	63,28	31,82	-	-	-	-	-
S482	175,2	58,33	63,8	8,41	118,1	175,37	58,75	-	8,17	114,45	175,1	58,31	63,84	8,24	115,2	-	-	-
G483	174,1	45,32	-	8,48	113,2	174,48	45,57	-	8,22	110,51	-	45,33	-	8,32	110,5	45,307	8,42	110,76
K484	177,1	56,28	32,82	8,27	123,2	176,92	56,58	32,75	7,96	119,59	-	56,17	-	-	-	-	-	-
G485	174,1	45,3	-	8,53	112,3	174,19	45,64	-	8,25	108,47	-	45,25	-	8,37	109,6	-	-	-
R486	176,8	56,16	30,65	8,28	123,1	175,21	56,37	30,7	7,89	119,03	176,7	56,21	30,99	8,12	120,3	-	-	-
G487	173,9	45,15	-	8,51	112,3	176,65	45,44	-	8,13	107,91	173,8	45,14	-	8,35	109,5	-	-	-
L488	177,3	54,99	42,33	8,19	124	176,99	55,02	42,19	7,77	120,07	177,1	55,03	42,31	8,01	121,3	-	-	-
Q489	175,4	55,68	-	8,44	123,5	175,19	55,84	29,39	8,09	119,03	175,2	55,62	-	8,26	120,5	-	-	-
H490	-	53,02	-	8,51	122,2	171,44	53,21	28,66	7,97	117,54	-	53,72	-	8,17	120,7	53,082	8,418	119,56
P491	-	-	-	-	-	-	-	-	-	-	-	-	-	-	-	-	-	-
P492	-	-	-	-	-	-	-	-	-	-	-	-	-	-	-	-	-	-
P493	176,9	62,65	31,84	-	-	-	-	-	-	-	176,9	62,64	31,88	-	-	-	-	-
V494	176,8	62,59	32,51	8,32	123,3	-	62,28	32,74	7,9	118,96	176,7	62,62	32,3	8,13	120,5	62,775	8,265	120,66
G495	174,2	45,15	-	8,59	115,4	174,3	45,32	-	8,31	111,31	174,1	45,11	-	8,44	112,6	45,244	8,522	112,6
S496	-	58,39	63,84	8,27	118	174,84	58,58	63,9	8,05	114,99	174,6	58,38	63,87	8,09	115,3	58,496	8,168	115,51
Q497	175,7	55,79	29,33	8,58	124,7	175,81	56,08	28,86	8,35	121,43	175,7	55,81	29,43	8,45	122	55,911	8,484	121,85
E498	176,2	55,87	29,77	8,44	124,2	176,28	56,24	30,06	8,24	120,33	176,4	56,43	30,64	8,3	121,7	55,752	8,337	121,05
G499	-	44,46	-	8,35	112,9	171,36	44,41	-	7,99	109,34	-	44,41	-	8,18	109,9	44,485	8,229	110,1
P500	177	62,95	32,11	-	-	-	-	-	-	-	176,9	62,88	32,09	-	-	62,824	-	-
K501	176,1	55,98	33,07	8,48	123,9	175,77	56,01	32,93	8,23	121	-	55,75	33,02	8,32	121,5	55,976	8,481	120,48
D502	-	51,77	-	8,35	124,4	174,34	51,62	40,91	8,16	121,21	-	-	-	8,17	122,2	-	-	-
P503	177,3	63,73	31,97	-	-	-	-	-	-	-	177,5	63,77	32,23	-	-	-	-	-
E504	176,5	56,25	28,73	8,41	121,4	176,49	56,75	28,91	8,16	117,31	177	56,87	29,61	8,33	118,9	-	-	-
A505	177,7	53,1	18,79	8,06	126	177,33	52,54	19,25	7,76	122,27	177,9	53,41	18,84	7,88	122,9	-	-	-
W506	176,2	57,08	29,22	7,86	120,9	175,62	57,02	29,5	7,66	119,35	176,2	57,29	29,12	7,67	117,5	-	-	-
S507	173,6	57,9	63,66	7,86	119,2	-	57,88	64,39	7,69	116,92	173,7	58,08	63,66	7,65	115,7	-	-	-
R508	-	53,84	-	7,97	125,9	-	55,27	-	7,96	121,9	-	53,91	-	7,75	123	-	-	-
P509	-	63,02	32,06	-	-	-	-	-	-	-	176,9	63,15	-	-	-	-	-	-
Q510	176,9	56,52	28,94	8,59	123	-	-	-	-	-	-	56,52	28,98	8,42	120,1	-	-	-
G511	174,1	45,38	-	8,56	112,5	-	45,58	-	8,14	108,17	173,9	45,28	-	8,35	109,5	-	-	-
W512	175,9	57,93	29,48	7,97	123,4	176,41	58,06	29,32	7,76	120,6	175,8	57,73	29,59	7,8	120,5	-	-	-
Y513	174,9	57,52	38,51	7,75	123	175,51	58,65	38,15	7,57	118,58	174,8	57,66	38,53	7,59	120,3	-	-	-
N514	174,8	53	38,62	8,13	122,4	175,17	53,34	-	7,9	119	-	52,89	-	7,95	119,8	-	-	-
H515	174,2	55,31	28,63	8,39	122,1	174,48	56,02	28,16	7,98	118,67	-	-	-	-	-	-	-	-
S516	174,7	58,59	63,78	8,4	119,5	174,91	58,63	-	8,1	115,6	174,8	58,62	63,76	-	-	-	-	-
G517	173	45,23	-	8,46	113,9	173,31	45,41	-	8,21	110,98	172,9	45,32	-	8,27	111,1	-	-	-
K518	-	57,36	-	7,94	128,1	181,31	57,5	33,42	7,66	125,57	-	57,41	-	7,69	125,6	-	-	-

Table S2. Secondary Cα chemical shifts of RIPK3 with corresponding error estimates based on signal-to-noise ratio analysis.

Assignment	ΔCα	σ(Cα)
G387	-1,918	0,00633
S388	-0,103	0,00317
S389	-0,04	0,00144
S391	0,15	0,00478
M392	-0,2	0,00144
A393	-0,115	0,00140
Q397	-0,09	0,00249
T398	-0,121	0,00192
E400	-0,689	0,00488
T401	-0,189	0,00182
S402	-0,074	0,00188
T403	0,044	0,00510
F404	0,49	0,00756
R405	0,151	0,00469
N406	-0,127	0,00345
Q407	-0,302	0,00308
M408	-0,224	0,00366
P409	0,158	0,00361
S410	0,046	0,00153
P411	0,404	0,00366
T412	-0,406	0,00171
S413	0,039	0,00098
T414	-0,418	0,00094
G415	-0,125	0,00094
T416	-0,235	0,00101
P417	-0,1	0,00533
S418	0,029	0,00169
P419	0,169	0,00868
G420	-0,16	0,00189
R422	-0,106	0,00249
G423	-0,008	0,00225
N424	-0,293	0,00269
Q425	-0,172	0,00145
G426	0,143	0,00249
A427	-0,157	0,00411
G483	-0,243	0,00372
H490	-0,368	0,00756
V494	0,218	0,00335
G495	-0,16	0,00313
S496	-0,046	0,00166
Q497	-0,189	0,00182
E498	-1,067	0,00155
G499	-0,272	0,00260
P500	-0,281	0,00478
K501	-0,389	0,00263
